# B7 family protein glycosylation: Promising novel targets in tumor treatment

**DOI:** 10.3389/fimmu.2022.1088560

**Published:** 2022-12-06

**Authors:** Linlin Xiao, Xiaoyan Guan, Mingli Xiang, Qian Wang, Qian Long, Chaoyi Yue, Lulu Chen, Jianguo Liu, Chengcheng Liao

**Affiliations:** ^1^ Department of Orthodontics II, Affiliated Stomatological Hospital of Zunyi Medical University, Zunyi, China; ^2^ Oral Disease Research Key Laboratory of Guizhou Tertiary Institution, School of Stomatology, Zunyi Medical University, Zunyi, China; ^3^ School of Medicine and Technology, Zunyi Medical University, Zunyi, China

**Keywords:** glycosylation, PD-L1, PD-L2, B7-H3, B7-H4, B7-H6, PD-1

## Abstract

Cancer immunotherapy, including the inhibition of immune checkpoints, improves the tumor immune microenvironment and is an effective tool for cancer therapy. More effective and alternative inhibitory targets are critical for successful immune checkpoint blockade therapy. The interaction of the immunomodulatory ligand B7 family with corresponding receptors induces or inhibits T cell responses by sending co-stimulatory and co-inhibitory signals respectively. Blocking the glycosylation of the B7 family members PD-L1, PD-L2, B7-H3, and B7-H4 inhibited the self-stability and receptor binding of these immune checkpoint proteins, leading to immunosuppression and rapid tumor progression. Therefore, regulation of glycosylation may be the “golden key” to relieve tumor immunosuppression. The exploration of a more precise glycosylation regulation mechanism and glycan structure of B7 family proteins is conducive to the discovery and clinical application of antibodies and small molecule inhibitors.

## Introduction

Changes in protein glycosylation are a pivotal regulatory part of tumor progression and directly affect cell growth, survival, tumor immune escape and final metastasis (1). Tumor-related crucial glycoproteins (such as EGFR (2, 3), CD44 (4), E-cadherin (5), TGF-β receptor (6, 7), CA199 (8) and MUC-1 (9, 10)), glycan abundance and structural changes profoundly affect tumor cell fate and patient prognosis. Most immune checkpoints are membrane glycoproteins, and increasing attention has been given to how protein glycosylation changes affect the tumor immune microenvironment (11) (12) (13). Programmed cell death ligand 1 (PD-L1) belongs to the B7 protein family and binds to its receptor programmed death receptor 1 (PD-1) on activated T cells to suppress antitumor immunity by counteracting T-cell activation signals (14). The role of glycoprotein PD-L1/PD-1 glycosylation in tumor immune regulation has been extensively studied (15–17). B7 family members also include B7-1, B7-2, PD-L2, B7-H2, B7-H3, B7-H4, B7-H5, BTNL2, B7-H6, B7-H7 and Ig-like domain-containing receptor 2 (ILDR2). These proteins and their receptors play critical roles in cell proliferation, cytokine secretion and tumor immune microenvironment regulation (18–21). B7-1 (22), PD-L2 (23), B7-H3 (24), B7-H4 (25), and B7-H6 (26, 27) were confirmed to be modified by glycosylation. However, current evidence suggests that glycosylation may not be required for the function of B7-1 (22) and B7-H6 (26, 27), and the significance of glycosylation for B7-1 and B7-H6 proteins is largely unknown. Therefore, the significance, mechanism and possibility of targeted therapy of PD-L1, PD-L2, B7-H3, B7-H4 and B7-H6 glycosylation are explored in this review.

## Significance, mechanism and possible use of PD-L1 glycosylation as a therapeutic target

**Figure 1:**
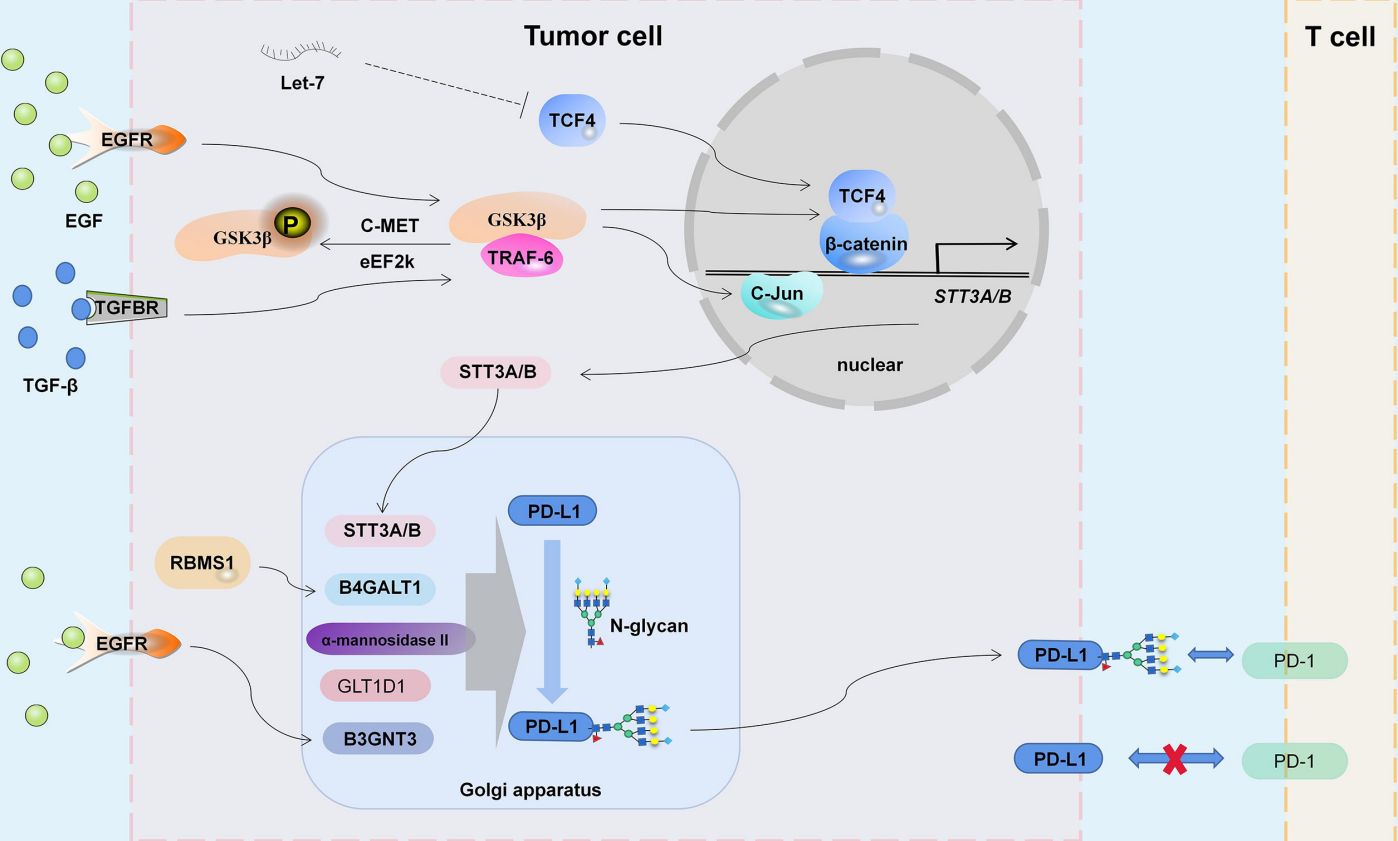
The mechanism of PD-L1 glycosylation. EGFR, TGF-β Signaling and noncoding RNA Let-7 participate in the regulation of STT3A/B expression by C-Jun and TCF4/β-catenin complex. Glycosyltransferases STT3A/B, B4GALT1, α-Mannosidase II, GLT1D1 and B3GNT3 regulate the glycosylation abundance of PD-L1, thus changing the tumor immune microenvironment.

The PD-L1 protein consists of an immunoglobulin V-like domain (IgV, F19-T127), an immunoglobulin C-like domain (IgC, P133-V225), a transmembrane domain (TM, T239-F259) and an intracellular short tail domain (R260-T290) (28, 29), and is affected by a variety of posttranslational modifications, including phosphorylation, glycosylation, palmitoylation, acetylation and ubiquitination (30). There are four N-glycan sites in the extracellular domain of PD-L1: N35, N192, N200 and N219 (31), and these N-glycans are critical to the stability of the PD-L1 protein (30, 31). PD-1 and PD-L1 interact through the large hydrophobic surface of their respective Ig-like V-domains, while the Ig-like C2 domain of PD-L1 has no contact with PD-1 (32). The interaction between PD-L1 and PD-1 also requires glycosylation (33). PD-1/PD-L1 binding can induce tumor-specific T-cell apoptosis by inhibiting T-cell activation and is currently one of the most important immunotherapeutic targets (34). The glycosylation site of PD-L1 may not be easily bound by antibodies (35, 36), and understanding the specific mechanism of PD-L1 glycosylation (**Figure 1**) is of practical significance for targeted therapy.

### STT3 mediates PD-L1 N-glycosylation

Oligosaccharide transferases (OSTs) generate N-glycosylated proteins by transferring oligosaccharides in lipolinked oligosaccharides (LLOs) to the asparagine residues of the Asn-Xaa-Ser/Thr receptor sequence (37). The mammalian OST subtypes carry STT3A or STT3B catalytic subunits for co-translation or post translation N-glycosylation modification (37). STT3A-OST and STT3B-OST are highly correlated, but there are important differences in their catalytic mechanisms and speed (38–40). The role of STT3A/B in tumor progression has been studied (41–43), and it plays an important role in the N-glycosylation of PD-L1 (44).

At the initial stage of tumor cell metastasis, epithelioid tumor cells transform into mesenchymal-like cells (EMT), resulting in loss of cell-to-cell contact, increased mechanical mobility, and invasion of the surrounding matrix (45). At the same time, the tumor immune microenvironment is also changing (46). The molecular relationship between EMT and tumor immune escape is being explored (47). For example, miR-200/ZEB1-mediated EMT progression can promote PD-L1 expression and subsequent CD8^+^ T-cell suppression (48). In addition, as an EMT transcription regulator, β-Catenin can also regulate the expression of PD-L1 (49–52). Hsu et al. (53) found that the TGF-β-driven β-catenin/TCF4 complex activates STT3A/B expression on the promoter of the STT3 subtype. In addition, TGF-β1 also promotes c-Jun binding to the promoter of STT3A and regulates STT3A expression at the transcriptional level (54). Early studies have shown that the two STT3 isomers act on peptides sequentially to maximize the efficiency of N-glycosylation (38). In triple-negative breast cancer (TNBC), nasopharyngeal carcinoma and HNSCC, STT3A/B increased the level of PD-L1 N-glycosylation and protein stability (53–55). The β-catenin inhibitor KYA1797K can downregulate the expression of STT3A/B, thereby inhibiting PD-L1 glycosylation and immune escape of colon CSCs (56). However, the difference in STT3A- and STT3B-mediated N-glycan modification and how they are regulated remain to be fully elucidated, and distinguishing the differences in PD-L1 N-glycosylation induced by STT3A and STT3B may provide ideas for more precise targeted therapy.

### EGFR/GSK3β mediated PD-L1 glycosylation and ubiquitination

Glycogen synthase kinase 3β (GSK3β) is a serine/threonine protein kinase originally identified as a regulator of glycogen metabolism (57, 58), which is widely believed to be associated with tumors, embryonic development, liver injury and aging (59). GSK3β induces the phosphorylation-dependent proteasome degradation of Snail, Mcl-1, SIRT7, GFI1, CRY1 and EZH2, leading to mesenchymal epithelial transformation, chemotherapy sensitivity, apoptosis and chromosome stability of cancer cells (60–65). Recently, the focus of research in this area has been strictly on decreasing the stability of the PD-L1 protein by inactivating GSK3β (phosphorylation at the T180, S9 and S184 sites), thereby regulating T-cell-mediated tumor immunity (66–70). Inactivation of GSK3β may stabilize the PD-L1 protein by increasing PD-L1 ubiquitination (31). However, the EGF/EGFR pathway not only inactivates GSK3β (31) but also promotes PD-L1 glycosylation (31, 71) and ubiquitination (72). In addition, Li et al. (31) ound that GSK3β binds to phosphorylated, nonglycosylated PD-L1 (N192, N200 and N219 residues are required), which may block PD-L1 glycosylation, and the glycosylation of N192, N200 and N219 antagonizes the interaction between PD-L1 and GSK3β. Furthermore, GSK3 is involved in the regulation of β-catenin (73), and GSK3β/β-catenin/STT3 may be one of the pathways that regulates PD-L1 glycosylation (53). Therefore, the EGFR/GSK3β pathway is required for PD-L1 protein stability and PD-L1/PD-1 interface maintenance.

### B3GNT3 mediates PD-L1 glycosylation

β-1,3-n-acetylglucosamine aminotransferase 3 (B3GNT3) is a type II transmembrane protein in the Golgi apparatus (74) that can form extended core 1 oligosaccharides (75). The relationship between B3GNT3 and tumor immunosuppression is being explored. B3GNT3 overexpression inhibits CD8^+^ T-cell infiltration in pancreatic cancer and promotes tumor progression (76). A metabolism-related gene pair index (MRGPI) study showed that the high expression of B3GNT3 and low expression of HSD17B6 may have a synergistic reaction in the immune escape of lung adenocarcinoma through the PD-1/PD-L1 pathway (77). The immunohistochemical data of 145 cases of primary lung adenocarcinoma also showed that the expression of B3GNT3 was closely and positively correlated with the expression of PD-L1 and EGFR mutation (78). B3GNT3 also regulates L-selectin ligand function, lymphocyte transport and T-cell homing (75). Li et al. (71) found that B3GNT3 induced by EGF can increase glycosylation at the N192 and N200 sites (poly LacNAc) of PD-L1 and promote PD-L1/PD-1 binding. In addition, B3GNT3 can activate NF-κB signaling (79). Considering the important role of NF-κB in tumor immunity (80, 81), B3GNT3 may regulate tumor immunity through multiple pathways.

### B4GALT1 mediates PD-L1 galactosylation

Seven members of the β4-galactosyltransferase (B4GALT) family have different biological functions due to differences in receptor specificity, tissue distribution, and temporal expression (82). B4GALT1 is the main enzyme responsible for the transfer of UDP-galactose residues to terminal N-acetylglucosamine residues in Golgi-processed glycoproteins (83), and its expression is involved in galactosylation of IgG, CDK11^p110^ and other proteins (84–88). In addition, B4GALT1 can also regulate the expression of glycans on proteins through the JAK signaling pathway (89, 90). B4GALT1 is overexpressed in pathological processes, such as inflammation and proliferation of cancer cells, which makes targetting this enzyme in anticancer therapy possible (91, 92). B4GALT1 expression was positively correlated with PD-L1 and CTLA4 expression in bladder cancer (93). In TNBC, RNA binding motif single strand interacting protein 1 (RBMS1) positively regulates B4GALT1 expression, which is related to the inhibition of inflammation and PD-L1-mediated antitumor immunity (94). Mechanistically, RBMS1 increases the stability of B4GALT1 mRNA and promotes B4GALT1-mediated PD-L1 galactosylation in N-glycans, thereby reducing PD-L1 protein degradation (94).

### *MAN2A1* mediates PD-L1 glycosylation

*The mannosidase α class II member 1 (MAN2A1)* gene encodes α-mannosidase II, which can transform the precursor high mannose type N-glycans into mature complex structures and is a key enzyme for N-glycan biosynthesis (95). The *MAN2A1* and *MAN2A2* genes are widely expressed in the human body at a relatively high level (96). The single deletion of *MAN2A1* or *MAN2A2* will leads to a relatively mild and organ-specific phenotype, but the simultaneous deletion of both genes will leads to embryonic death and complete lack of complex N-glycans (97). Shi et al. (98) knocked out MAN2A1 in tumor cells, and simple/precursor and heterozygous N-glycans increased, while complex N-glycans decreased. Therefore, the lack of α-mannosidase II weakened PD-L1/PD-1 binding-mediated T-cell immunosuppression (98). The α-mannosidase inhibitor swainsonine makes tumors sensitive to anti-PD-L1 therapy (98). However, α-mannosidase II is highly similar in structure to lysosomal α-mannosidase, and coinhibition of these two proteins was produced when targeting α-mannosidase II and resulted in severe side effects, weakening the potential of α-mannosidase II as a therapeutic target (99).

### GLT1D1 is involved in PD-L1 glycosylation

The glycosylation process mainly involves the sequential action of different glycosyltransferase families, and their expression and function are strictly regulated in each cell (100). In addition to STT3, B3GNT3, B4GALT1 and α-mannosidase II, which are involved in PD-L1 glycosylation (54, 71, 94, 98), other glycosyltransferases involved or possibly involved in PD-L1 glycosylation have also been explored. Glycosyltransferase 1 containing domain 1 (GLT1D1) is highly upregulated in incurable B-cell non-Hodgkin’s lymphoma subtypes and early relapsed diffuse large B-cell lymphoma (101) and may be associated with poor prognosis of colon cancer (102) and multiple myeloma (103). GLT1D1 expression is positively correlated with glycosylated PD-L1 levels in B-cell non-Hodgkin’s lymphoma, and high GLT1D1 expression is associated with poor prognosis of patients (101). GLT1D1 plays an important role in the N-glycosylation and stability of the PD-L1 protein. The downregulation of GLT1D1 reduces the glycosylation of the PD-L1 protein, leading to an increase in cytotoxic T-cell infiltration in the tumor microenvironment (101). However, GLT1D1 is an insufficiently studied glycosyltransferase, and its specific modification form is unclear.

### Glycosylation is critical for PD-1 stability and its binding to PD-L1

Approximately 20%-90% of protein N-glycans on the cell surface are generated by core fucosylation, which is catalyzed by α-1,6 fucosyltransferase (FUT8) (104, 105). FUT8-mediated core fucosylation modification of the TGF-β receptor and E-cadherin, PD-1 and α3β1 integrin proteins is vital for their function (106). In fact, the four N-glycans of PD-L1 are highly core fucosylated (31), but the significance of FUT8-mediated PD-L1 N-glycan core fucosylation has not been discussed. However the loss of core fucosylation significantly enhanced the ubiquitination of PD-1, which led to the degradation of PD-1 in the proteasome (107). Highly N-glycosylated PD-1 is widely expressed in T cells and is the key to maintaining the stability and cell surface localization of PD-1 protein, especially the glycosylation at the N58 site, which is necessary to mediate its interaction with PD-L1 (16, 108). Monoclonal antibodies STM418 (108), camrelizumab (17), mAb059c (109) and penpulimab (110) specifically target glycosylated PD-1 and have a high binding affinity for PD-1, effectively inhibiting PD-L1/PD-1 binding and enhancing anti-tumor immunity. In addition, adenine base editor (ABE) induces the conversion from a-t to g-c at specific sites, changes the coding sequence of the N74 residue of *PDCD1* in CAR-T cells, downregulates the expression and glycosylation of PD-1 in CAR-T cells, and enhances the cytotoxicity *in vitro* and *in vivo* (111).

### Inhibiting PD-L1 glycosylation to improve tumor immune infiltration

Antibodies targeting the immune checkpoint receptor PD-1 or its ligand PD-L1 are used to treat various types of cancer, and can significantly improve the survival of patients (112). However, drug resistance in tumor immunotherapy forces us to look for more effective inhibitors. Direct/indirect inhibition of PD-L1 glycosylation is a potential strategy to achieve therapeutic effects. The antibodies STM004 and STM108 constructed by Li et al. (71) effectively block the interaction of PD-L1/PD-1. STM108 recognizes the N192 and N200 glycosylation sites, and the amino acid cross-linking is closer to the C-terminal domain of PD-L1 (Y81, K162 and S169); STM004 recognizes the N35 glycosylation site, and amino acid cross-linking is relatively close to the N-terminal domain of PD-L1 (Y56, K62 and K75) (71). STM108 can specifically recognize the B3GNT3 mediated poly LacNAc part on N192 and N200 glycosylation sites of PD-L1, and induce PD-L1 internalization and degradation (71). Metformin can improve the effect of immune checkpoint inhibitor therapy (113), changing the glycan structure of PD-L1 by activating AMPK, thus promoting the degradation of PD-L1 and thereby blocking the immunosuppressive signal (114). 2-deoxyglucose (2-DG) can be used as a glucose analog to reduce PD-L1 glycosylation and reverse the immunosuppression induced by polyadenosine-diphosphate-ribose polymerase (PARP) inhibitor in TNBC (115, 116). In addition, D-mannose can also activate the AMPK pathway to phosphorylate PD-L1 at the S195 site, leading to abnormal glycosylation and degradation of PD-L1 (117).

Resveratrol is a kind of polyphenolic stilbene that is found in grapes, mulberries, peanuts, rhubarb and several other plants and is used to treat diabetes, obesity, cardiovascular disease, neurodegeneration and cancer (118). Resveratrol can affect the expression of PD-L1/PD-1 and the subcellular localization and posttranslational modification of PD-L1 (119). Resveratrol regulates the N-glycosylation modification of PD-L1 by inhibiting α-glucosidase/α-mannosidase, a mannose-rich abnormal glycosylated form of PD-L1 that inhibits binding to PD-1 (120). In addition, resveratrol promotes PD-L1 dimerization by interacting with the inner surface of PD-L1 (111), but dimerized PD-L1 can also bind to PD-1 and regulate T-cell toxicity (121, 122). Poor pharmacokinetics and low potency seem to be the two main bottlenecks of resveratrol (123). However, resveratrol combined with a PD-L1 inhibitor can not only significantly promote the infiltration of CD8^+^/CD4^+^ T cells but also significantly inhibit the number of Treg cells and MDSCs at the same glycolysis level (124).

Inhibitors of glycosyltransferase associated with PD-L1 glycosylation have been developed. The small molecule OST inhibitor such as NGI-1 can inhibit the activities of STT3A and STT3B at the same time (125, 126). Puschnik et al. (127) found that 12 inhibitors such as me-3,4-depostatin, hispidin, myricetin and piceatannol can inhibit B4GALT1 activity. And α-Mannosidase II can be inhibited by pyrrolidine compounds and salacinol family compounds (128). These inhibitors are potential drugs to improve tumor immunosuppression mediated by PD-L1 glycosylation. However, the role of these glycosyltransferase inhibitors in the treatment of tumors and PD-L1 deglycosylation still needs to be further explored.

## Structure and significance of PD-L2 glycosylation

PD-L1 and PD-L2 (B7-DC) are the two main ligands of PD-1 (129). The binding affinity of PD-L2 to PD-1 is 2-6 times higher than that of PD-L1 to PD-1 (130). In addition, PD-L2 is also the combination partner of repulsive guidance molecule b (RGMb), which improves respiratory tolerance (131). Similar to PD-L1, PD-L2 has an N-terminal IgV domain and a membrane proximate IgC domain. However, the PD-L1 IgV domain contains the C’ and C″β chains, while in the PD-L2 IgV domain the C’ and C″β chains are replaced by a flexible C-D ring (132). The IgV domain of PD-L2 binds to PD-1, and its glycosylation structure regulates its affinity for PD-1 (133). The stability of the PD-L2 protein is related to the N-glycosylation sites N157, N163 and N189 but not N64 (23). The N64 glycan is located in the C-D ring region of PD-L2, and its glycosyl structure and solubility increase the dynamic characteristics of the C-D ring region. Furthermore, the affinity of PD-L2 for PD-1 can be improved by removing the N64 glycan (119). In addition, the “pocket” formed by N64 may be the binding site of PD-L2/PD-1 affinity drugs (134). However, the glycosylation significance of PD-L2 at the N10 and N43 sites has not been clarified (135).

PD-L2 is expressed on immune cells, dendritic cells and other types of hematopoietic and non hematopoietic cells (136) (137) (138). PD-L2 and PD-1 inhibit T-cell proliferation mediated by the T-cell receptor (TCR) and cytokine production (139). Although the frequency or intensity of PD-L2 expression may not be as high as that of PD-L1 in most tumors (140), PD-L2 can be expressed without PL-L1 expression in some specific tumors. PD-L2 is expressed or strongly expressed in 51.7% of esophageal adenocarcinomas, while PD-L1 is expressed in 2% of esophageal adenocarcinomas (141). PD-L2 was expressed in 62.7% of HNSCCs (more than twice as many as for PD-L1), and 61.4% of HNSCCs were PD-L1-negative (142). In addition, Xu et al. (23) found that PD-L2 was N-glycosylated and upregulated in tumor tissues of HNSCC patients resistant to cetuximab. Deglycosylation inhibited the expression of PD-L2 in colorectal cancer cells (143). In HNSCC, the STAT3 pathway activates FUT8-mediated PD-L2 glycosylation, which stabilizes the PD-L2 protein by blocking ubiquitin-dependent lysosome degradation, thereby promoting its combination with PD-1 and immune escape (23). Moreover, glycosylated PD-L2 forms a complex with EGFR, which leads to the activation of EGFR/STAT3 signaling and reduces the binding affinity of cetuximab for EGFR (23).

## Significance of B7-H3 glycosylation

As a type I transmembrane protein, B7-H3 consists of an extracellular domain, a transmembrane domain and a short intracellular domain. The human B7-H3 protein has two isomers determined by the extracellular domain: 4IgB7-H3 and 2IgB7-H3. 4IgB7-H3 consists of two pairs of identical IgV-like domains and IgC-like domains, 2IgB7-H3 consists of one pair of identical IgV-like domains and IgC-like domains, and 4IgB7-H3 is more common in human cells (144). B7-H3 is abnormally highly expressed in lung (145), ovarian (146), glioblastoma (147), colorectal (148), gastric (149), prostate (150), urothelial (151), brain (152), pancreatic (153), breast (154), cholangiocarcinoma (155), hepatocellular (156), oral (157) and renal (158) cancer cells and can be induced to be expressed on antigen presenting cells (APCs), including dendritic cells (DC) and macrophages (159). In the tumor microenvironment, B7-H3 inhibits CD4^+^ and CD8^+^ T cell responses by inhibiting IFN-γ, IL2, IL-10 and IL-13 (160) and promotes the immune escape of tumor cells (161, 162). In addition, B7-H3 also has nonimmunogenic effects, promoting tumor cell migration, invasion, angiogenesis, drug resistance, and EMT and regulating cell metabolism (144). These results make B7-H3 a potential target for tumor therapy.

**Figure 2:**
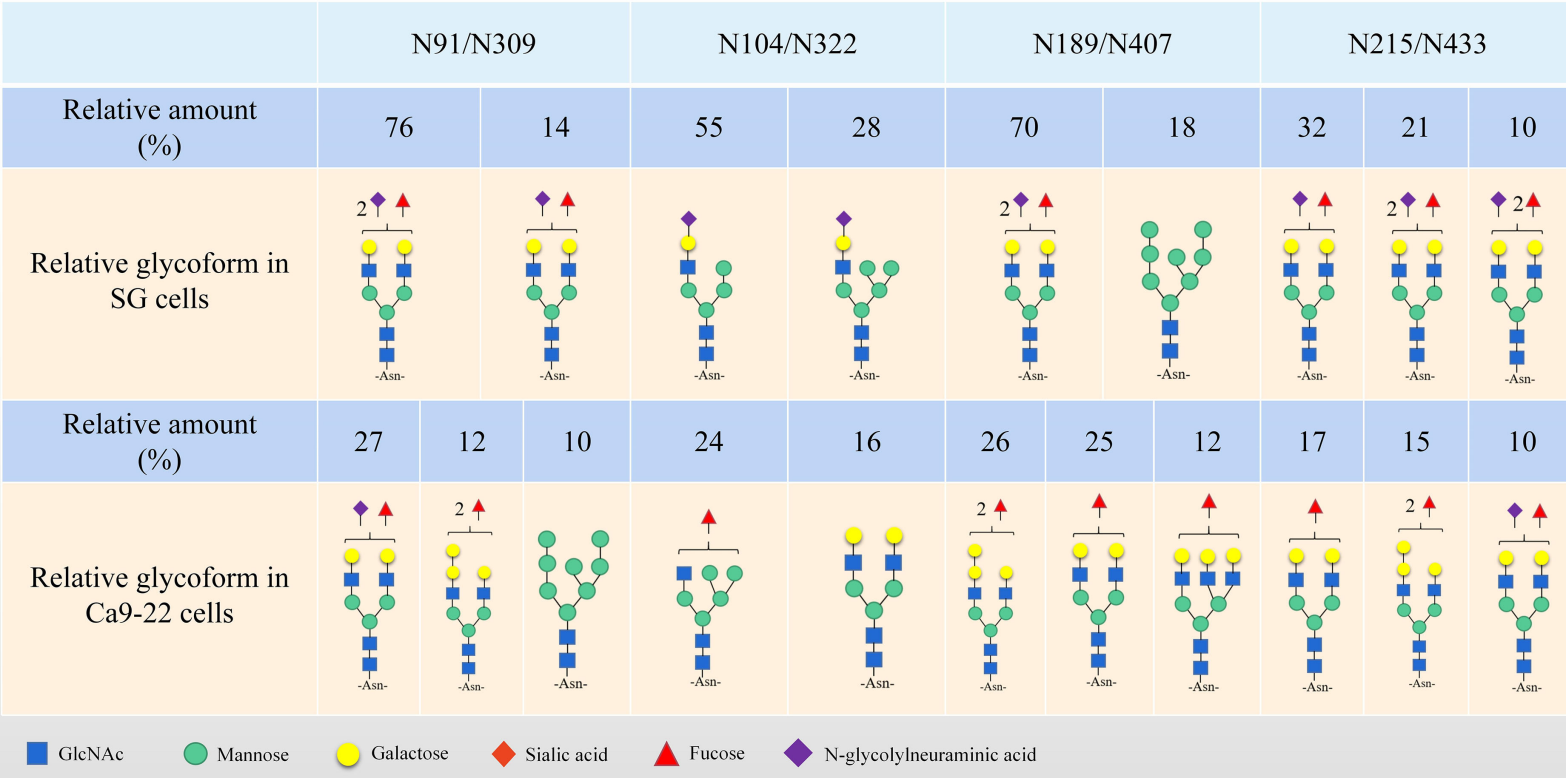
Site-specific representative glycans of B7-H3.

B7-H3 is a highly glycosylated protein. Human B7-H3 protein can have eight N-glycan sites, N91, N104, N189, N215, N309, N322, N407 and N433 (**Figure 2**) (24, 163). Chen et al. (157) found that the glycan of B7-H3 in an OSCC cell line contains a more diversified N-glycan structure and a terminal α-galactose, and the glycan rich structure of B7-H3 may allow it to play an important role in the progression of oral cancer. In esophageal squamous cell carcinoma (ESCC), the increased level of B7-H3 protein N-glycan fucosylation promotes the occurrence and development of tumors (164). Huang et al. (24) found that the N-glycosylation of B7-H3 at the NXT motif site is related to protein stability and triple-negative breast cancer (TNBC) immunosuppression. In breast cancer, there are more than 140 N-glycan core fucosylated glycoproteins mediated by FUT8 (165, 166). Knockout of FUT8 inhibits the immunosuppressive function mediated by N-glycosylated B7-H3 in TNBC cells (24). Moreover, the combination of the core fucosylation inhibitor 2F-Fuc and anti-PD-L1 can enhance the therapeutic effect of B7-H3-positive TNBC (24).

## Significance of B7-H4 glycosylation

The B7-H4 (also known as B7x, B7S1 or VTCN1) protein consists of 282 amino acids, including an extracellular domain, a large hydrophobic transmembrane domain and an intracellular domain composed of only two amino acid residues (132). The B7-H4 protein has the overall structure of a type I transmembrane protein. Similar to other B7 family members, its extracellular domain has a pair of Ig-like domains. However, the homology of this protein with other B7 family members is only approximately 25% (132). Different from other B7 family members with restricted mRNA expression, *B7-H4* mRNA is widely expressed in the brain, heart, kidney, liver, lung, ovary, pancreas, placenta, prostate, skeletal muscle, skin, thymus and uterus (167). Although *B7-H4* mRNA is widely expressed in normal human cells, the distribution of B7-H4 protein on the surface of normal cells is rare (168). However, the B7-H4 protein is highly expressed in human tumors and is associated with the clinicopathological features of patients (157). The expression of B7-H4 in gastric (169, 170), breast (171, 172), lung (145), prostate (173), pancreatic (174), bladder (175), colorectal (176), ovarian (177), renal (178), urothelial (179), esophageal (180), and gallbladder (181) cancers is associated with tumor size, primary tumor grade, TNM stage, low survival rate, drug resistance and a decreased number of tumor-infiltrating T cells. B7-H4 inhibits the proliferation, cell cycle progression and cytokine production of CD4^+^/CD8^+^ T cells (168, 182), attenuates the inflammatory response, and enables tumor cells to evade the immune system (132, 183).

**Figure 3:**
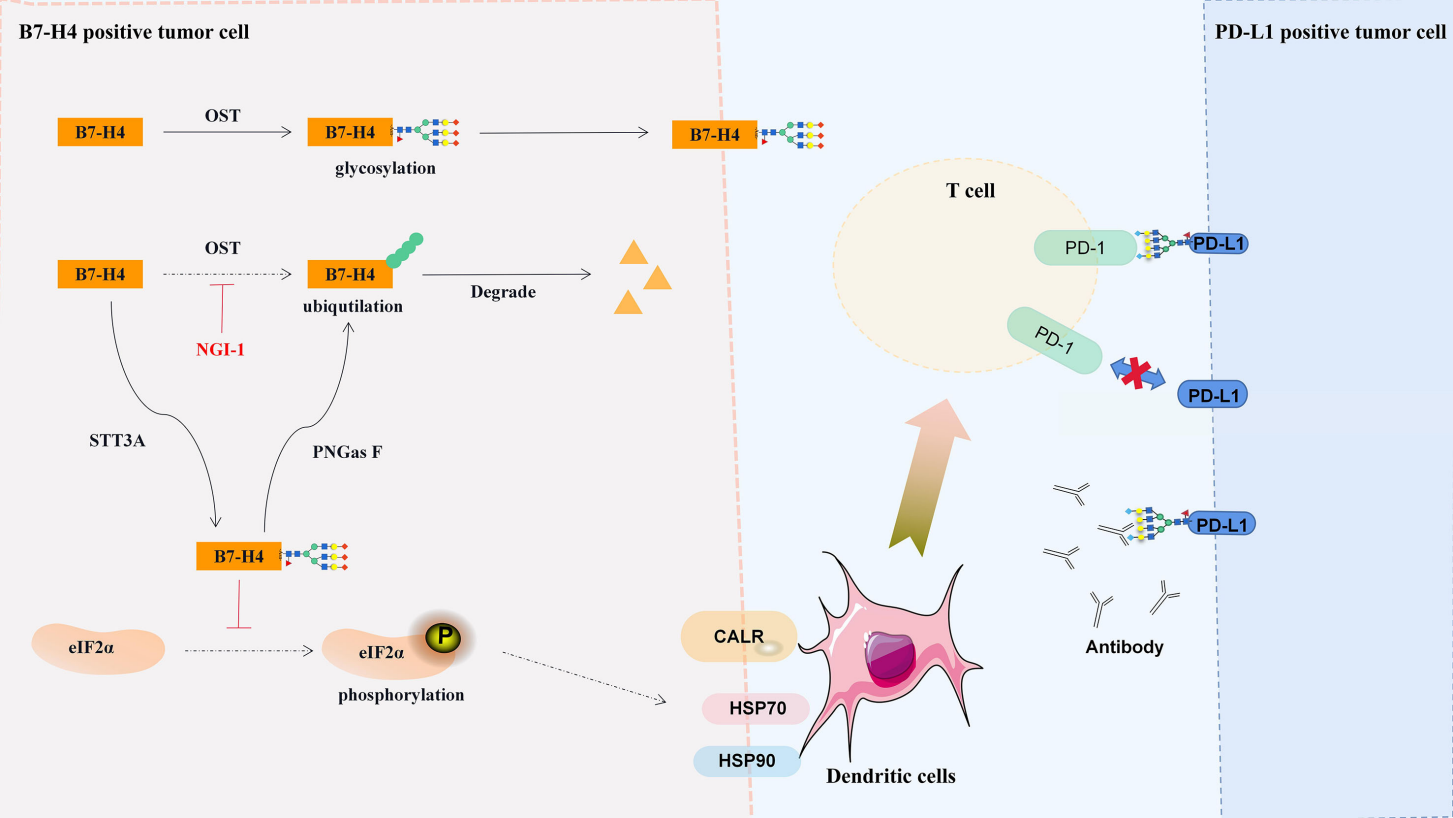
Regulation and significance of B7-H4 glycosylation. Glycosylated B7-H4 inhibits the phosphorylation of eIF2α and dendritic cell recruit, and deglycosylation lead to the ubiquitination and degradation of B7-H4. CALR, calreticulin; HSK70, heat shock protein 70; HSK90, heat shock protein 90.

Salceda et al. (184) found that the highly glycosylated B7-H4 protein was overexpressed in most serous ovarian cancers and breast cancers but was hardly expressed in normal tissues, mucous or low-grade malignant ovarian cancers. The accumulation of glycosylated B7-H4 expression in immunocompetent breast cancer was negatively correlated with the expression of PD-L1 (25), and similar results were also found in glioma (185), lung (186) and pancreatic (187) cancer. Therefore, B7-H4 may be the key to treating PD-L1-negative cold tumors, and B7-H4 glycosylation sites are potential therapeutic targets. In 293T cells, B7-H4 has five N-glycans (N112, N140, N156, N160 and N255) and two ubiquitination sites (K146 and K138) (25). Glycosylation can stabilize the structure of the B7-H4 protein, blocking the phosphorylation of eIF2α, reducing the exposure of calreticulin, and thus inhibiting the immunogenicity of cancer cells (**Figure 3**) (25). Moreover, the B7-H4 ubiquitination site can only be detected in the presence of PNGas F, and glucosyltransferase STT3A/UGGG1-mediated N-glycosylation at the asparagine site interferes with the ubiquitination of lysine residues (**Figure 3**) (25).

## Glycosylation mediates the interaction between B7-H6 and NKp30

B7-H6 is a type I transmembrane protein consisting of two extracellular IgG-like domains (IgV and IgC), an α-helical transmembrane domain, and homologous C-terminal sequences of population specific antigen (GAG) proteins (26). The B7-H6 C-terminal sequence has a variety of signal motifs, including ITIM, SH-2 and SH-3 binding motifs, which can trigger signal transduction after binding with natural killer protein 30 (NKp30) (26, 27) and activate NK cells and cytokine secretion (188). The closest structural homolog of B7-H6 is PD-L1, and both are highly glycosylated glycoproteins (26, 27). However, although the B7-H6 protein has five predicted N-glycosylation sites, Skořepa et al. (189) found that the removal of the B7-H6 N-glycan did not affect its crosslinking with NKp30. In contrast, NKp30 glycosylation modification is essential for the interaction between NKp30 and B7-H6 (190). The removal of the N68 glycosylation site of NKp30 reduced its affinity for the B7-H6 ligand, while the removal of the N42 glycosylation site of NKp30 almost completely eliminated its binding to the B7-H6 ligand (179). Targeting NKp30 to treat tumors has proven to be effective, and CAR-T cells expressing chimeric NKp30 receptors can destroy B7-H6^+^ cells (191, 192).

## Conclusion

The binding of antibodies to PD-L1 was affected by the degree of protein glycosylation. Deglycosylation of PD-L1 enhances binding to 28-8, CAL10, CAL10, SP142, atezolizumab and SP142 MAbs (193, 194) but is not conducive for binding to avelumab (195). Targeting PD-L1 glycosylation promotes its degradation and inhibits the binding of some antibodies to PD-1 but also increases the therapeutic effect of some antibodies. The complex network composed of STT3A/B, EGFR/GSK3β, B3GNT3, B4GALT1, α-mannosidase and GLT1D1 regulates PD-L1 glycosylation and participates in PD-L1 stabilization and binding to PD-1 (**Figure 1**). Glycosylation is also important for the protein function and stability of PD-L2, B7-H3 and B7-H4. Blocking or targeting PD-L1/2 and B7-H3/4 protein glycosylation may be an important supplement for tumor immunotherapy. A more comprehensive study of the glycosylation modification of the B7 protein family will reveal a new direction for the translational application of glycobiology in tumor immunotherapy.

## Author contributions

CL, LX, XG and JL contributed to the conception and design of the review. CL and LX wrote the manuscript. YG, JL and QW validate the manuscript. MX, QL, CY and LC contributed to draw the figures. All authors contributed to the article and approved the submitted version.
